# The “Big Two” in Hiring Discrimination: Evidence From a Cross-National Field Experiment

**DOI:** 10.1177/0146167220982900

**Published:** 2021-03-06

**Authors:** Susanne Veit, Hannah Arnu, Valentina Di Stasio, Ruta Yemane, Marcel Coenders

**Affiliations:** 1 DeZIM-Institut, Berlin, Germany; 2 WZB Berlin Social Science Center, Berlin, Germany; 3 Utrecht University, Utrecht, The Netherlands; 4 Sociaal en Cultureel Planbureau (SCP), Den Haag, The Netherlands

**Keywords:** stereotypes, hiring discrimination, field experiment, ethnic minorities

## Abstract

We tested whether signaling warmth and competence (“Big Two”) in job applications increases hiring chances. Drawing on a field experimental data from five European countries, we analyzed the responses of employers (*N* = 13,162) to applications from fictitious candidates of different origin: native candidates and candidates of European, Asian, or Middle-Eastern/African descent. We found that competence signals slightly increased invitation rates, while warmth signals had no effect. We also found ethnic discrimination, a female premium, and differences in callbacks depending on job characteristics. Importantly, however, providing stereotype signals did not reduce the level of ethnic discrimination or the female premium. Likewise, we found little evidence for interactions between stereotype signals and job demands. While speaking against the importance of “Big Two” signals in application documents, our results highlight the importance of group membership and hopefully stimulate further research on the role of in particular *ethnic* stereotypes for discrimination in hiring.

The world is on the move. Not only because of globalization and the increasing ease of mobility but also because of violent conflicts and wars, many people—voluntarily or involuntarily—leave their home countries and migrate to other countries, with Western democracies being popular destination countries. These migration flows have altered the ethnic composition of the population in Western democracies. In 2014, about 55 million first- and second-generation immigrants^1^ resided in the European Union (EU), which equals 18% of all EU residents^2^ (Eurostat, 2016).

Given the high share of residents with foreign roots, ethnic minorities’ successful integration in European societies is of high priority. Regarding labor market integration (e.g., labor market activity, likelihood to be employed, and wages), however, a comprehensive body of empirical literature points to ethnic penalties. With some variation across countries, immigrant groups, and specific indicators of labor market success, overall, immigrants and their descendants tend to do worse on the labor market than native residents (Algan et al., 2010; Auer et al., 2019). Poorer language skills and lower levels of education among immigrants contribute to this gap, but in most studies, a gap remained also when controlling for human capital differences (Gorodzeisky & Semyonov, 2017; Heath & Cheung, 2007; Heath et al., 2008). This residual gap might indicate that immigrants and their descendants perform worse on the labor market partly because of discrimination from employers. Indeed, recent meta-analyses and reviews of correspondence tests support this view by providing robust evidence of ethnic and racial discrimination in hiring, which seems to be ubiquitous (Baert, 2018; Bertrand & Duflo, 2017; Neumark, 2018; Zschirnt & Ruedin, 2016) and persistent over time (Heath & Di Stasio, 2019; Quillian et al., 2017).

To explain ethnic and racial discrimination in hiring processes, researchers often refer to classic economic theories by contrasting statistical discrimination theory (Aigner & Cain, 1977; Arrow, 1971; Phelps, 1972) and taste-based discrimination theory (Becker, 1957; but see also Bartoš et al., 2016, on attention discrimination; Rooth, 2010, on implicit discrimination; and England, 1992, on error discrimination). The central difference between statistical and taste-based discrimination theories is that according to the former, employers are risk-averse and base their hiring decisions on perceived productivity- and competence-related differences at the group level while the latter explains discrimination by personal preferences of the employer (or of customers and co-workers) that are unrelated to productivity, such as dislike of certain ethnic groups or ethnic homophily and the preference for members of the in-group (Byrne, 1997; McPherson et al., 2001).

Interestingly, very similar dimensions are central in so-called “Big Two” models of social perception and stereotypes, like the Stereotype Content Model (SCM) by Fiske and Cuddy (Cuddy et al. 2008, 2009; Fiske et al., 2002). According to the SCM, warmth and competence are fundamental dimensions in how we perceive others, because humans are evolutionarily predisposed to judge strangers’ intentions to harm or help (warmth) and their capacity to act in line with their intentions (competence). The conceptual overlap between discrimination theories and fundamental stereotype content dimensions suggests that the perceived warmth and competence of job candidates might be important in hiring decisions.

In this article, we build upon and try to bridge two different strands of research literature. On one hand, field experiments on ethnic and racial discrimination in the labor market, which have provided strong empirical evidence of discrimination. These studies have mostly been conducted within economics and sociology and have paid less attention to the role of stereotypes about warmth and competence. On the other hand, the “Big Two” models of social perception and stereotypes, originating from social psychology, are hardly applied to theories of taste-based and statistical discrimination and to hiring more generally (but see Agerström et al., 2012, for a noteworthy exception). We draw upon “Big Two” models of social perception to draw hypotheses about the impact of signaling warmth and competence in job applications, and the degree to which this varies by gender and ethnic origin of applicants, as well as different types of jobs.

We present the results of cross-nationally harmonized correspondence tests of hiring discrimination (Lancee, Birkelund, Coenders, Di Stasio, Fernández Reino, Heath, Koopmans, Larsen, Polavieja, Ramos, Thijssen, et al. 2019). This study was simultaneously conducted in five European countries (Germany, the Netherlands, Norway, Spain, and the United Kingdom) and considers employer responses to more than 13,000 fictitious applications. The design of the study is innovative as it includes a wide variety of ethnic origin groups and female and male job candidates, which allows us to assess the role of warmth and competence signals across different job applicants and types of jobs.

## The “Big Two”: Warmth and Competence

According to “Big Two” models of social judgment (Abele & Wojciszke, 2013, 2014; Brambilla et al., 2012; Cuddy et al., 2008; Fiske et al., 2002; Rosenberg et al., 1968; Wojciszke, 2005), “two basic content dimensions underlie the judgments of social targets, including groups, cultures, other individuals, and the self” (Brambilla et al., 2011, p. 135): *warmth* (sometime referred to as communion, sociability, or morality) and *competence* (also: agency, capacity, or industriousness). Borrowing from evolutionary theory, the relevance of perceived warmth and competence is assumed to result from the importance for survival of the decision of whether to approach or to avoid strangers (Fiske et al., 2007). Warmth attributions reflect the impression that a person or group is trustworthy, helpful, cooperative, and has good intentions. One may approach strangers from a group that has such qualities, while members of cold and competitive groups may pose a danger and should be avoided. Likewise, ascribed competence reflects the perception that a person or group is able to act upon their intentions (no matter whether these intentions are good or evil)—in contrast to persons or groups who are perceived to be low in competence.

The SCM (Cuddy et al., 2008; Fiske et al., 2002) belongs to the “Big Two” models of social perception and stereotypes and identifies warmth and competence as the two fundamental stereotype content dimensions (but see also Koch et al., 2016, or Abele et al., 2016). Stereotypes are beliefs about the typical characteristics of the members of a group (Yzerbyt, 2016). Stereotypes guide information processing and social judgments, in particular under time pressure or cognitive overload (Dijksterhuis & Van Knippenberg, 2006). According to the SCM, warmth is the primary dimension, because warmth judgments are made more quickly and have greater overall impact on attitudes. Moreover, the SCM proposes that groups’ status in society predicts competence stereotypes, whereas perceived intergroup competition predicts warmth stereotypes. For example, large immigrant groups that compete with natives for housing and jobs may be perceived as threatening and therefore colder, while immigrant groups that stand out as particularly poor, educationally alienated, and part of the under-class are perceived as less competent. Finally, the SCM proposes that stereotypes are often ambivalent rather than clearly positive or negative, meaning that most social groups are perceived as being high on one dimension but low on the other dimension, that is, either “warm but incompetent” (e.g., mothers) or “cold but competent” (e.g., rich people).

Many previous studies on the SCM have focused on gender. Empirical research has repeatedly shown that women are perceived as being higher in warmth but lower in competence than men (Fiske, 2017; Rudman & Glick, 1999). This ambivalent stereotype is a source of disadvantage for females on the labor market, where being perceived as competent is essential for success. In line with this reasoning, previous meta-analyses on gender discrimination in hiring have confirmed a penalty for female job candidates (Koch et al., 2015; Olian et al., 1988). This gender penalty, however, is rather small in size and tends to disappear, and even reverse, in applications for female-dominated jobs, as shown in both simulated and real hiring situations (Davison & Burke, 2000; Riach & Rich, 2002).

Comparably fewer studies have focused on ethnic and racial minorities or immigrants. Lee and Fiske (2006) found that the “generic” immigrant is typically perceived as being low on both warmth and competence; whereas specific immigrant groups (e.g., German immigrants) often receive ambivalent stereotypes (see also Reyna et al., 2013). Moreover, they proposed that the content of stereotypes about specific immigrant groups depends on the stereotype about the respective *national origin* group and the socio-economic status of the respective *immigrant* group in the receiving society. Finally, a few studies investigated the content of ethnic stereotypes more into detail (Eagly & Kite, 1987; Ghavami & Peplau, 2013), but they were all conducted in the United States and it is not clear whether their findings can be generalized to the European context. Moreover, it is still unknown to what extent the SCM can explain ethnic and racial discrimination in hiring.

## Studying Discrimination in Hiring

There is a high and ever-growing number of field experiments on ethnic discrimination, also known as correspondence tests (Baert, 2018; Bartkoski et al., 2018; Neumark, 2018; Quillian et al., 2017; Rich, 2014; Zschirnt & Ruedin, 2016), that provide causal evidence of hiring discrimination. To this end, researchers send application documents of fictitious job candidates to real vacancies and register employers’ responses. By keeping all characteristics other than the characteristic of interest (e.g., ethnic background) constant, differences in response rates between groups provide causal evidence of discrimination.

While being very successful in demonstrating the existence of discrimination in hiring, correspondence tests have been less successful in disentangling the reasons that lead employers to discriminate. In classical economic literature, discrimination is described as resulting either from assumed productivity differences at the group level and risk aversion (statistical discrimination, see Aigner & Cain, 1977; Guryan & Charles, 2013; Phelps, 1972) or from preferences that are unrelated to productivity, such as ethnic homophily and the preference for similarity (Byrne, 1997; McPherson et al., 2001) or the dislike of certain ethnic groups (taste-based discrimination, see Becker, 1957). This distinction ties in with the two fundamental dimensions proposed by the SCM. While statistical discrimination is influenced by a groups’ ascribed *competence*, taste-based discrimination reflects a negative social relation that is best described as the desire to avoid contact with dissimilar, competitive, and threatening—in other words, cold—groups.

## Bridging the Gap: The “Big Two” and Discrimination in Hiring

In line with the conceptual closeness between the “Big Two” dimensions and classic explanations for discrimination in hiring, there is substantial research on the role of *gender* stereotypes (i.e., warm but incompetent women and competent but cold men) and *gender* role congruity (i.e., the fit between gender roles and work-related roles) for *gender* discrimination in hiring (Koch et al., 2015). At the same time, however, there are surprisingly few empirical studies that relate the role of *ethnic* stereotypes and stereotype consistent or inconsistent information in application documents to *ethnic* discrimination in hiring. A noteworthy exception is a study by Agerström et al. (2012, see also Rattan et al., 2019) on the Swedish job market. Focusing on male job candidates with either Swedish-sounding or Arab-sounding names, they investigated the consequences of conveying a warm and social (or a cold and self-involved) personality and of conveying high competence and a strong work ethic (or a relaxed attitude toward work combined with a strong preference for a good work–life balance). They found that candidates with Arab-sounding names had always significantly lower chances of receiving an invitation for a job interview than candidates with Swedish names, unless one compared invitation rates between “cold and incompetent Swedish” job candidates and “warm and competent Arab” job candidates. Assuming that fellow Swedes are stereotypically rather warm and competent while Arabs are stereotypically rather cold and incompetent (Lee & Fiske, 2006), this finding suggests that providing stereotype-inconsistent information may reduce ethnic penalties. However, this strategy makes minorities just comparable with “bad” majority members who admit being self-involved and incompetent (see also Arai et al., 2016). Moreover, the generalizability of this finding to other countries, minority groups, or occupations has not yet been tested.

## Hypotheses

In this article, we set out to answer the following research questions:

**Research Question 1:** What is the impact of signaling warmth and competence in job applications on the likelihood that applicants receive a positive response from employers?
**Research Question 2:** To what extent does this vary by gender and ethnic origin of job applicants, and across different types of occupations?

### Stereotype Content Signals

First, we build upon the SCM to develop hypotheses about the main effects of warmth and competence signals in job applications. We expected recruiters and employers to respond to signals of warmth and competence when screening application documents (Cuddy et al., 2011). As warmth is the primary dimension in person perception (Brambilla et al., 2012; Brambilla & Leach, 2014; Wojciszke et al., 1998) and has a clearly positive valence, we expected invitation rates to increase for job candidates who signal a warm personality. Within hiring decisions, however, skills and work motivation are perhaps even more important. While the SCM predicts warmth to generally take primacy, “within organizational context, competence judgments may again take primacy” (Cuddy et al., 2011, p. 77).

Another argument for the relative importance of (positive) competence information in hiring decisions draws on the diagnostic value of positive or negative warmth and competence information. In our study, we assess the impact of including *positive* information about warmth and competence in job applications. Note that we did not include *negative* information, as this is unrealistic; in real life, job applicants try to convince employers of their qualities by means of some form of “impression management” in their application materials. Cuddy et al. (2011) proposed that positive competence information is generally more consequential than negative competence information, while negative warmth information is more consequential than positive warmth information. The reason for this asymmetry partly lies in the diagnostic value of warmth and competence information (Tausch et al., 2007). Whereas one can feign warmth by intentionally behaving in a warm manner, it is difficult to behave competently without being competent. Consequently, one salient contradiction of warmth often suffices to change warmth impressions to the worse. For competence, by contrast, repeated failures are necessary to change a positive competence impression to the worse and one salient demonstration of competence can be sufficient to regain the image of a competent individual.

Hence, we expected a positive main effect of the competence signal (H1a) as well as of the warmth signal (H1b), and we expected the positive effect of the competence signal to be larger than the positive effect of the warmth signal (H1c):

**Hypothesis 1a (H1a):** Signaling competence increases job candidates’ chances of receiving a positive response from employers.
**Hypothesis 1b (H1b):** Signaling warmth increases job candidates’ chances of receiving a positive response from employers.
**Hypothesis 1c (H1c):** In relative terms, signaling competence more strongly increases job candidates’ chances of receiving a positive response from employers than signaling warmth.

### Stereotype Content Signals and Gender

How employers react to warmth and competence signals in application documents, however, may also vary with the stereotype of the group a job candidate belongs to and with the fit between stereotype and stereotype signal. Previous research has shown that stereotypes about social groups indeed affect personnel selection decisions, to the disadvantage of job candidates who belong to groups that are stereotypically depicted as incompetent (e.g., women: Koch et al., 2015; older people: Krings et al., 2011; disabled people: Louvet, 2007; cancer survivors: Martinez et al., 2016). According to Fiske et al. (2002), however, ambivalent stereotypes are very common. Stereotypically competent groups tend to be stereotypically depicted as cold (Cuddy et al., 2011: contrast effects), because a surplus on one dimension is often compensated for with the assumption of a deficit on the other dimension (Kervyn et al., 2009). Men, for example, are stereotypically depicted as competent but cold while women are stereotypically depicted as warm but incompetent (Koch et al., 2015). Likewise, specific immigrant groups are often perceived to be either warm but incompetent or competent but cold, like, for example, German immigrants (Lee & Fiske, 2006).

Stereotypes not only describe how members of specific social groups are perceived but also how they are *supposed* to be (Ellemers, 2018). According to Bodenhausen (1988; see also Maio et al., 1996), stereotype activation leads to biased information processing, with stereotype-consistent information receiving more attention than stereotype-inconsistent information (selective information processing; see also Maio et al., 1996).^3^ In line with this reasoning, Cuddy and colleagues (2011) proposed that, within organizations, evaluators credit members of stereotypically competent groups for success much more than members of stereotypically incompetent groups, because they perceive information on past success to be diagnostic for the former but not for the latter. In case of failure, they predict the opposite pattern to occur. For members of stereotypically competent groups, failures might be interpreted as bad luck, whereas for members of stereotypically incompetent groups, failures are perceived as diagnostic for a person’s “true” incompetence. Likewise, the warmth stereotype may affect how evaluators interpret instances of nice or nasty behaviors: For members of stereotypically warm groups, nice behaviors are seen as an indication of disposition and nasty behaviors as a slip caused by the situation. The opposite is true for members of stereotypically cold groups.

We therefore expected different effects of warmth and competence signals on employer responses, depending on the fit between the signal that was provided and the stereotype of the group to which a job candidate belongs. As women are stereotypically depicted as high in warmth but low in competence whereas men are stereotypically depicted as high in competence but low in warmth (Koch et al., 2015), we expected stronger effects of warmth information on employer responses to applications from female (vs. male) job candidates and stronger effects of competence information on responses to applications from male (vs. female) job candidates (see also Swim & Sanna, 1996):

**Hypothesis 2a (H2a):** Signaling warmth more strongly increases the likelihood of receiving a positive employer response for female job candidates than it does for male job candidates.
**Hypothesis 2b (H2b):** Signaling competence more strongly increases the likelihood of receiving a positive employer response for male than for female job candidates.

### Stereotype Content Signals and Ethnic Origin

In a similar vein, ethnic group membership is associated with stereotypes. Previous research suggested that people perceive in-group members and people who are like themselves as warm and competent while out-group members are often subject to negative stereotypes. Generic immigrants, for example, are stereotypically cold *and* incompetent (Lee & Fiske, 2006). Specific immigrant groups, however, often receive ambivalent stereotypes (either warm but incompetent or competent but cold), depending on the national stereotype and immigrant groups’ status in the receiving society. Even though the content of stereotypes about national groups slightly differs across countries and depending on the target group, there is surprisingly high consensus about the stereotype content of groups belonging to specific world regions (Cuddy et al., 2009; Linssen & Hagendoorn, 1994): From a Western perspective, Europeans and U.S. citizens are stereotypically depicted as high in warmth and competence, East Asians are stereotypically depicted as high in competence but low in warmth, and people from the Global South are stereotypically depicted as low in competence and warmth. Considering the information processing mechanism in relation to job candidates’ ethnic background, we therefore expected warmth and competence signals to be particularly beneficial for job candidates who belong to ethnic groups that are stereotypically competent or warm, respectively:

**Hypothesis 3a (H3a):** Signaling warmth more strongly increases the likelihood of a positive employer response for native and immigrant job candidates of European origin than for immigrant job candidates of Asian background or for immigrants of Middle-Eastern or African origin.
**Hypothesis 3b (H3b):** Signaling competence more strongly increases the likelihood of a positive employer response for native and immigrant job candidates of European and Asian origin than for immigrant job candidates of Middle-Eastern or African origin.

### Stereotype Content Signals and Job Characteristics

Finally, we were interested in the interplay between job characteristics and stereotype content signals. We focused on the level of customer contact (low vs. high), as a warm personality might be particularly relevant in jobs that involve frequent interactions with customers, and on the required level of education (either high or medium to low), as high competence might be particularly important in high-skilled occupations. We thus expected employers to reward warmth signals more strongly in occupations that involve high (vs. low) levels of customer contact while we expect them to reward competence signals more strongly in high-skilled occupations than in low- to medium-skilled occupations:

**Hypothesis 4a (H4a):** Signaling warmth more strongly increases the likelihood of positive employer responses in occupations that involve high levels of customer contact than in occupations that involve low levels of customer contact.
**Hypothesis 4b (H4b):** Signaling competence more strongly increases the likelihood of a positive employer response in high-skilled occupations than in low-skilled ones.

## Method

### Data

We tested our hypotheses drawing on a unique data set on hiring discrimination that was gathered within the framework of a large international research project on the labor market integration of migrants (GEMM Project: Growth, Equal Opportunities, Migration & Markets, Horizon 2020 research and innovation program, Grant Agreement No 649255).

The correspondence test was conducted between summer 2016 and spring 2018 in Germany, Netherlands, Norway, Spain, and the United Kingdom (see the technical report: Lancee, Birkelund, Coenders, Di Stasio, Fernández Reino, Heath, Koopmans, Larsen, Polavieja, Ramos, Thijssen, et al., 2019). Each employer received only *one* fictitious application. An unpaired design was necessary to accommodate many treatments (e.g., migration background, ethnicity, religion) and treatment conditions (e.g., various countries of origin). In addition, an unpaired design was also preferable for ethical reasons as it reduces the burden to employers and it minimizes the risk that employers could get suspicious when receiving very similar applications and might change their behavior purely for fear of being part of an audit (Larsen, 2020).

The design of the correspondence test was cross-nationally harmonized. By the term *harmonized*, we refer to the fact that we included the same treatments and applied comparable procedures simultaneously in all five countries. However, we deviated from the shared experimental protocol whenever necessary to comply with country-specific application standards (for more details, see Lancee, Birkelund, Coenders, Di Stasio, Fernández Reino, Heath, Koopmans, Larsen, Polavieja, Ramos, Soiné, et al., 2019; Lancee, Birkelund, Coenders, Di Stasio, Fernández Reino, Heath, Koopmans, Larsen, Polavieja, Ramos, Thijssen, et al., 2019). For example, in Germany, job candidates must send copies of their school and job training certificates when applying for a job, while this is not the case in most other European countries. We therefore added such documents when applying for jobs in Germany. For the same reason, we did not add photos to resumes in Norway or the United Kingdom, where this would be frowned upon, but we did so when applying for jobs in Spain and the Netherlands or in Germany.^4^

### Sample

Detailed descriptions of the experimental design and procedure as well as of all experimental treatments are provided in the technical report and in the codebook of the larger project (Lancee, Birkelund, Coenders, Di Stasio, Fernández Reino, Heath, Koopmans, Larsen, Polavieja, Ramos, Soiné, et al., 2019; Lancee, Birkelund, Coenders, Di Stasio, Fernández Reino, Heath, Koopmans, Larsen, Polavieja, Ramos, Thijssen, et al., 2019). In what follows, we briefly introduce the variables and experimental treatments that are central to the present study.

In each country, the original sample consisted of 25% applicants from the native population and 75% immigrants originating from a large number of countries. Moreover, in each country, applicants from two sizable or historically well-established minority groups were oversampled (these applications amounting to 25% of the total sample): Turks and Lebanese in Germany, Moroccans and Turks in the Netherlands, Pakistani and Somali in Norway, Ecuadorians and Moroccans in Spain, and Nigerians and Pakistani in the United Kingdom. However, we only retained those oversampled groups that were included in the design of the correspondence test in all five countries (i.e., not Somalis and Ecuadorian). Of the migrant applicants in the sample, 50% were second-generation immigrants and 50% foreign-born who migrated at pre-school age. It can be safely assumed that their language fluency is on par with that of natives. All of them completed their education and training in the current country of residence, which rules out poor comparability in qualifications as a possible explanation for differences in callbacks.

We made several selections to the original sample. We focused only on the six occupations that were tested in all five destination countries: cook, store assistant, payroll and admin clerk, receptionist, software engineer, and marketing and sales representative (see Table 1).

**Table 1.** Observations by Study Country and Occupation.

Occupation	Germany	Netherlands	Norway	Spain	The United Kingdom	%
With little customer contact						
Software developer^a^	412	542	320	165	413	14
Payroll clerk^b^	411	638	300	547	777	20
Cook^b^	403	728	252	1,104	357	22
With much customer contact						
Sales representative^a^	422	549	488	170	526	16
Receptionist^b^	417	391	56	359	389	12
Store assistant^b^	413	444	205	556	408	15
Total	2,478	3,292	1,621	2,901	2,870	100

a Occupation with high qualification requirements. ^b^Occupation with medium to low qualification requirements.

Moreover, we limited our sample to 29 ethnicities that belong to the following four larger origin groups (see Table 2): natives (i.e., members of the national majority without foreign roots), immigrants of European origin (Western and Eastern Europe, including Russia), Asian origin (East and South-East Asia, including Pakistan), and MEA origin (Middle-East and Africa, including Turkey).^5^ Our final sample consisted of 13,162 applications sent in response to job openings in six occupations in five countries (see Tables 1 and 2).

**Table 2.** Observations by Countries and Origin Groups.

Native origins		Immigrant origins					
		Europe		Asia		Middle-East/Africa	
Germany	705	Germany	136				
Netherlands	982	Netherlands	183				
Norway	537	Norway	170				
Spain	958	Spain	171				
The United Kingdom	786	The United Kingdom	184				
		Albania	351	China	251	Egypt	234
		Bulgaria	324	India	232	Ethiopia	220
		France	225	Indonesia	221	Iran	235
		Greece	246	Japan	247	Iraq	253
		Italy	226	Pakistan	853	Lebanon	422
		Poland	349	South Korea	223	Morocco	945
		Romania	216	Vietnam	202	Nigeria	638
		Russia	214			Turkey	838
						Uganda	185
Total	3,968		2,995		2,229		3,970

### Measures and Experimental Treatments

#### Positive response

The dependent measure was *positive response*, a dummy variable indicating whether the employer signaled any interest in the job candidate (0 = “no,” 1 = “yes”). We registered all responses from employers, such as confirmations of receipt, invitations to interview, requests for a callback, and rejections. Thereafter, we identified the final response, which was either the *only* response ever received (e.g., outright rejection) or the *first* response that gave an idea of whether the application would be considered further (e.g., notification that the application had been shortlisted and was given serious consideration for the post). We interpret as positive response all requests to provide additional information, call the employer back, work on a trial basis or invitations to a job interview. We coded explicit rejections, non-responses, and confirmations of receipt that were not followed up as signs of a lack of interest from the employer.

#### Warmth and competence

We randomly assigned signals of a warm personality (warmth signal) and high occupational competence (competence signal) to both the resume and cover letter. The warmth and competence signals were positive self-descriptions, that were either present or absent. The assignment likelihood was identical for both signals, resulting in the following distribution: warmth (yes: 51%, no: 49%) and competence (yes: 50%, no: 50%). Combining both signals, the following distribution emerged: “warmth & competence” (25%), “warmth only” (25%), “competence only” (25%), and “neither warmth nor competence” (24%).

Warmth was signaled by a statement in the cover letter of the applicant that read as follows:

My colleagues and friends think I am a pleasant and warm person who gets along with people from all walks of life. I am a team player who values a positive work environment and that is why I am always friendly and attentive to other people’s needs. (For the verbatim formulation, see Lancee, Birkelund, Coenders, Di Stasio, Fernández Reino, Heath, Koopmans, Larsen, Polavieja, Ramos, Thijssen, et al., 2019)

Competence was signaled in a cover letter statement stressing competencies, achievements, and motivation, as well as by means of additional bullet points in the CV where the candidates listed additional responsibilities in their prior job. The statement read as follows:

My experience as [occupational title] has prepared me well to work under pressure. While taking on a wide variety of tasks and duties I have been able to show my ability to rise to challenges. I am a fast learner and I am always eager to develop new skills. My present employer has been very satisfied with my work and has passed more responsibilities on to me. For example, since last year I have been training the new [supervisees’ occupational title]. I am confident that I can bring the same level of high performance to your team.^6^

A post hoc validation study (see the Online Supplement) confirmed that the experimental stimuli had the intended effects and increased the perceived communion or agency of the applicants. The effects were significant, but rather small in terms of power. However, this is little surprising as we prioritized the plausibility and ecological validity of our treatments over maximal power by using only positive warmth and competence signals (because it is unrealistic that candidates would present themselves to employers as cold and incompetent in real job applications) and self-descriptions instead of reference letters (as including reference letters in job applications is not a common practice in some European countries).^7^

#### Gender

Job candidates’ gender (0 = “male,” 1 = “female”) was signaled by male or female names, respectively. In some countries, gender was also signaled by male and female job titles, by résumé photos, or by gender-specific connotations in the national language (e.g., in German).

#### Origin group

Job candidates’ country of origin was signaled in different ways. First, all job candidates had names that are typical for their countries of origin. Second, in the CV, all minority candidates listed the language of their country of origin together with the national language as first languages. Third, the country of origin was mentioned in the cover letter. Specifically, job candidates justified their application for a job outside their current city of residence based on the desire to move back to the region where they grew up and went to school. The cover letter contained one of the following statements, depending on job applicants’ migration status: (a) “I was born in [country of origin], but moved to [region of company] at the age of 6 and all my relevant education and training has been in [host country]” (for foreign-born minorities), (b) “My family is originally from [country of origin], but I was born in [region of company] and all my education and training has been in [host country]” (for second-generation immigrants), or (c) “I was born and raised in [region of company]” (for natives).^8^ In Germany and the Netherlands, the place of birth was also mentioned in the CV, reflecting country-specific application standards.

#### Qualification requirements and customer contact

We classified the six targeted occupations with respect to their qualification requirements (0 = “medium to low”: cook, payroll clerk, receptionist, and store assistant; 1 = “high”: sales representative and software developer) and the frequency of *customer contact* that can be expected (0 = “low”: cook, payroll clerk, and software developer; 1 = “high”: receptionist, store assistant, and sales representative).

#### Controls

Other job candidate characteristics that were varied (i.e., phenotype, religion, and grades) but fall outside the scope of this study were included as controls in all analyses (see Online Supplement Table S1). We also controlled for country using a set of dummy variables: 0 = “Germany,” 1 = “the Netherlands,” 2 = “Norway,” 3 = “Spain,” and 4 = “United Kingdom.”

### Empirical Strategy

We first explored the overall response pattern by conducting bivariate analyses, for the full sample and for subgroups (e.g., by gender, occupation, or study country), respectively. To test our hypotheses, we ran a series of multivariate linear regression models. We fitted *linear* probability models with robust standard errors despite our dichotomous dependent variable, because the results of linear regressions can more easily be interpreted and compared across models (Hellevik, 2009). We regressed the response received from employers on the warmth and competence signals, job candidates’ gender (male vs. female) and origin group (native vs. immigrant of either European, Asian, or MEA background), and the type of job (low- vs. high-skilled and requiring little vs. much customer contact), next to the control variables.

To demonstrate the robustness of our findings across model specifications, we re-ran all analyses as logistic and probit regression models. The pattern of results did not change (see Online Supplement Tables S3 and S4). In addition, we repeated all analyses adding probability weights to adjust for the unequal distribution of ethnic groups across countries and occupations (see Online Supplement Table S5). Again, most of the results remained unchanged. The very few aspects that did change are discussed below.

Second, we took into account potential biases in consequence of our rather rough regional grouping of origin group and repeated the main and moderation analyses with a more fine-grained regional grouping of origin groups (see Supplemental Figures S2 and S3).

Finally, we conducted a post hoc validation study with a German convenience sample to demonstrate the validity of our experimental manipulation in the application documents (for details, see the Online Supplement). We measured perceived communion and its subcomponents warmth and morality as well as perceived agency and its subcomponents competence and assertiveness by means of 20 descriptive adjectives that had been previously used to test the facets model (see Abele et al., 2016). Our analyses revealed that the inclusion of the warmth treatment had a significant positive effect on perceived communion and warmth, while the inclusion of the competence treatment had a significant effect on perceived agency, assertiveness, and competence. Importantly, the warmth signal had no effect on perceived agency and the competence signal had no effect on perceived communion. Thus, the validation study confirmed that our manipulation of warmth and competence was successful.

## Results

### Descriptive Results

**Figure 1.**
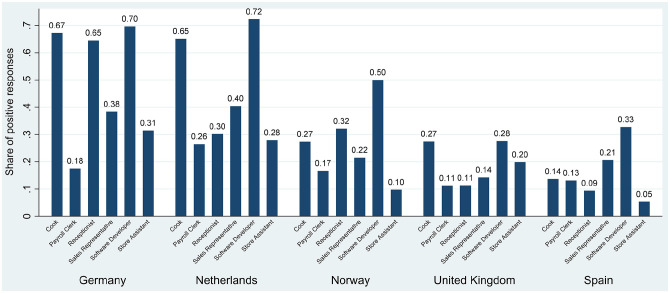
Response rates by occupation and country. *Note.* This bar plot provides the share of positive responses by job type and country (raw data, without any controls).

The distribution of the main variables of interest is shown in Online Supplement Table S1. Out of all job applications, 30% received a positive response from the employer, with strong variation across countries and occupations (see Figure 1). Whereas in Germany and the Netherlands almost every second application received a positive response, this rate was much lower in Norway (26%), in Spain (20%), and the United Kingdom (17%).

In general, the likelihood of receiving a positive response was particularly high for software developers and, in some countries, also for cooks and receptionists. For sales representatives, payroll clerks, and store assistants, by contrast, the likelihood of receiving a positive response was generally relatively low. Occupation-specific and national labor shortages are a plausible explanation for these differences.

### The Effect of Signaling Warmth and Competence

**Figure 2.**
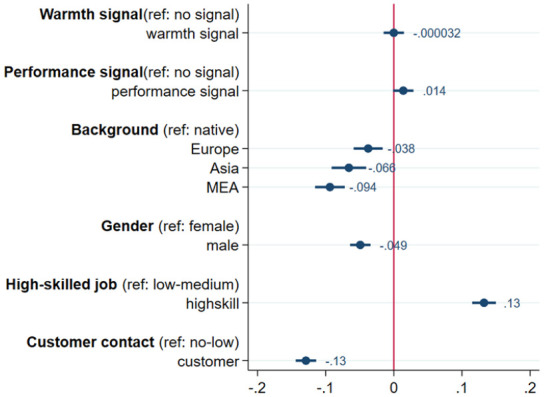
Main effects of applicant and job characteristics. *Note.* This coefficients plot shows regression coefficients and corresponding 90% and 95% confidence intervals that result from the linear regression of employer responses on gender, origin group, warmth and competence information while controlling for religion, grades, country, and occupation characteristics. MEA = Middle-Eastern/African.

Figure 2 provides the coefficients plot for the linear regression of employer responses on competence and warmth signals, job candidates’ origin group, gender, and job demands. Compared with standard applications, applications that placed more emphasis on job candidates’ competencies received slightly more favorable responses. The effect of competence is in line with expectations positive, but small (*b* = .01, *p* < .10; see also Online Supplement Table S2:1).^9^ By contrast, providing a warmth signal did not alter response rates (*b* = –.00 *ns*). Moreover, the interaction between signaling warmth and competence was not significant (see Online Supplement Table S2:2). While this finding remained unchanged when running logistic or probit regression models instead of linear ones, the effect of signaling competence was no longer significant after adding analytical weights to the regression to adjust for unequal cell sizes (*b* = .00, *ns*; see Online Supplement (Tables S3-S4).

Our results were thus partly supportive of H1a on a positive effect of signaling competence, but clearly contradicted H1b on a positive effect of signaling warmth. In addition, we rejected H1c, because the regression coefficient of competence was not significantly different from the coefficient of warmth; *F*(1, 13144) = 1.67, *p* = .196.^10^

In line with previous studies on ethnic discrimination in hiring, the likelihood of receiving a positive response was significantly lower for immigrant job candidates than it was for members of the national majority (see Online Supplement Table S2:1). The immigrant penalty ranged from four percentage points for minorities of European origin (*b* = –.04, *p* < .01) to seven percentage points for minorities of Asian origin (*b* = –.07, *p*<.01) and nine percentage points for job candidates originating from MEA countries (*b* = –.09, *p* < .01). In addition, across all occupations, employers significantly favored female job candidates over male ones (*b* = –.05, *p* < .01) and response rates were significantly higher in high-skilled jobs (*b* = .13, *p* < .01) and significantly lower in jobs with higher levels of customer contact (*b* = –.13, *p* < .01).

### Moderation by Gender, Origin Group, and Job Demands

**Figure 3.**
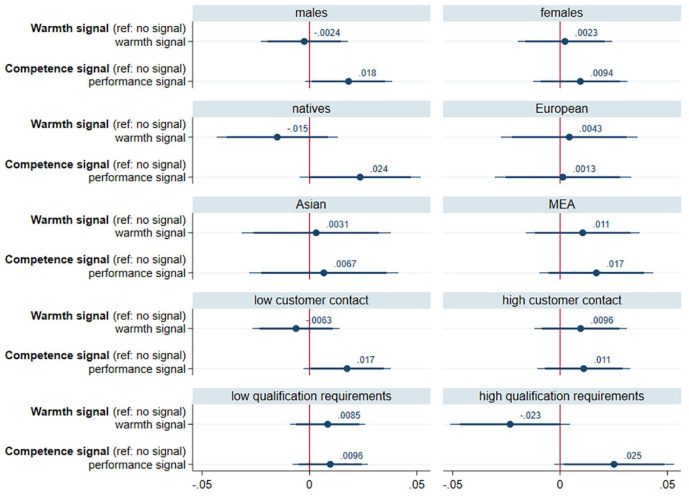
Results of separate regressions by job demands. *Note.* These two coefficient plots show the effects (with 90% and 95% confidence intervals) of warmth and competence signals on response rates in separate linear regressions for male and female job candidates, or job candidates who belong to different origin groups, and for occupations with low or high levels of customer contact and qualification requirements, respectively. All estimates result from linear regression models with robust standard errors and controls for job candidates’ religion, grades, and the country of study. MEA = Middle-Eastern/African.

Next, we tested whether the effect of signaling warmth and competence varied with characteristics of the candidate (i.e., gender and origin group) or of the job (see Figure 3). As the first row in Figure 3 illustrates, signaling warmth and competence had virtually the same effect for male and female job candidates. We thus rejected H2a and H2b (see also Online Supplement Table S2:3 and 4).

The second and third rows in Figure 3 (see also Online Supplement Table S2:5 and 6) show the effects of the stereotype content signals separately for our four origin groups: natives, European migrants, Asian migrants, and MEA migrants. We expected native job candidates to strongly benefit from both signals, especially in comparison with MEA migrants. For the warmth signal, however, we found the opposite pattern. For native job candidates, signaling warmth even had negative effects (by trend) on response rates and European immigrants benefited as much as other immigrants from signaling warmth. Signaling competence, by contrast, was indeed by trend positive for native job candidates. Against our expectation, however, it was also by trend positive for MEA immigrants and not at all beneficial for immigrants of European origin. Interaction analyses confirmed that there was no significant interaction between origin group and signaling competence, nor was there a significant interaction between origin group and signaling warmth (see Online Supplement Table S2:5 and 6). We therefore had to reject H3a and H3b.

When using more detailed origin categories (see Online Supplement Figures S1-2), the pattern of results slightly changed. For Central and Northern European immigrants, the pattern of results was very similar to the pattern for natives, while Southern European immigrants (who are stereotypically warm) profited from warmth more than any other group. South-East Asian immigrants, by contrast, profited from competence signals more than any other group. For all remaining origin groups (i.e., Eastern European, Eastern Asian, Southern Asian, MENA, and Sub-Saharan African), there were hardly any effects. Overall, these additional analyses provided some support for the origin-warmth hypothesis (Southern European immigrants), but no evidence in line with the origin-competence hypothesis.

Third, we explored whether the effects of stereotype content signals varied with job characteristics (i.e., qualification requirements and customer contact). Despite the strong main effects of job demands, none of the interactions with stereotype signals was statistically significant (Online Supplement Table S2:7 and 8). Neither H4a nor H4b received empirical support. *By trend*, however, the pattern of results was consistent with our hypotheses (see the last two rows in Figure 3)—in particular with respect to the role of warmth and competence signals in jobs with high qualification requirements.

Finally, we conducted exploratory analyses on the effects of warmth and competence signals for males and females of different national origin. As Online Supplement Figure S3 shows, the comparison between the different subgroups revealed a rather inconsistent pattern. Overall, the pattern of results fits our expectations best for native males. In jobs with low qualification requirements or little customer contact, they received a premium for competence signals while they were by trend penalized for signaling warmth.

### Do “Warm” and “Competent” MENA Migrants Catch Up With Native Job Candidates?

Last, we conducted additional analyses to replicate the findings by Agerström and colleagues (2012) that male Arab job candidates need to signal both warmth and competence to receive similar callback rates as male applicants with Swedish names who signal to be “cold” and “incompetent.” Note, however, the design differed between the two studies. In our study, the fictitious job candidates either did or did not signal warmth and competence, but they never signaled to be “cold” or “incompetent.”

**Figure 4.**
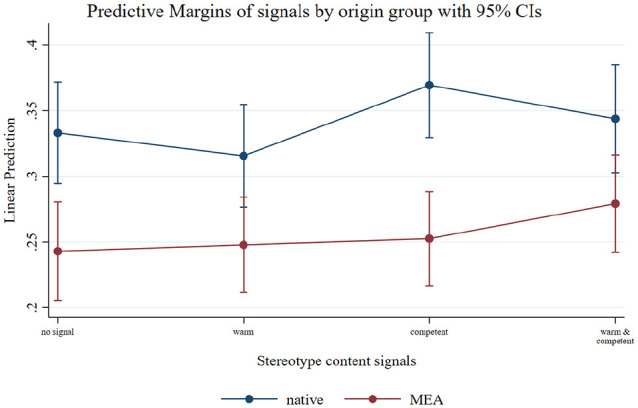
The effect of warmth and competence signals for native job candidates and job candidates originating in MEA countries. *Note.* To replicate the findings reported by Agerström et al. (2012), this margins plot shows the treatment effects of warmth and competence signals for native job candidates and job candidates originating in MEA countries (with 95% confidence intervals). The estimates result from linear regressions of employer responses on warmth and competence signals with robust standard errors, controlling for gender, customer contact, qualification requirements, job candidates’ religion, grades, and the country of study. MEA = Middle-Eastern/African.

To this end, we reduced our sample to observations from native males and males originating from MENA countries (i.e., Middle-Eastern and *North* African countries: Egypt, Iran, Iraq, Lebanon, Morocco, and Turkey). In addition to the penalty for MENA immigrants, we found that the treatment effects differed between the two groups. While male majority candidates were even slightly penalized for signaling warmth (alone or in combination with competence), male MENA minority candidates benefited most from simultaneously signaling warmth *and* competence (see Figure 4).

More precisely, even though male MENA migrants were on par with male native job candidates in most comparisons between treatments conditions, they hardly ever catched up with natives who signaled competence but no warmth. To catch up with them, male MENA migrants had to appear warm *and* competent. In sum, our results were similar, but not identical, to the results reported by Agerström and colleagues (2012).

## Summary and Discussion

We drew on a cross-nationally harmonized correspondence test to investigate the consequences of signaling warmth and competence in job applications in five European countries: Germany, the Netherlands, Norway, Spain, and the United Kingdom. On average, about one third of all applications received a positive response. However, response rates differed considerably between countries, occupations, and groups of job candidates. In relative terms, ascriptive characteristics of the job candidate had stronger effects on response rates than signals of warmth and competence. Our results thus illustrate the importance of group membership. Despite equal qualifications, male job candidates had about five percentage points lower chances of receiving a positive response than female candidates. This result is, at prima facie, surprising given the extensive literature of labor market discrimination to the disadvantage of women. It should be noted, though, that this female advantage is limited to female-dominated occupations and to women from the majority group, as we show in another study (Di Stasio & Larsen, 2020; see also Birkelund et al., 2019).

We also observed a pattern of ethnic discrimination that fits theory and previous empirical findings. Job candidates of foreign origin had significantly lower chances of receiving a positive response from employers than native job candidates (cf. Di Stasio et al., 2019; Veit & Thijsen, 2019). This gap amounted to about four percentage points for European minorities but increased up to nine percentage points for candidates of MEA descent, suggesting that employers follow an ethnic hierarchy when making hiring decisions.

The main goal of this study was to investigate whether signals of the “Big Two” of social perception, namely, warmth and competence, affect hiring decisions. We found very limited support for our hypotheses. For competence signals, the effect was positive but small, and it disappeared when also warmth was signaled or when analytical weights were added to the regression analysis. Moreover, the effects of warmth and competence did not vary with characteristics of job candidates or jobs. Hence, we found only very little support for theories on the primacy of stereotype-consistent information in cognitive processing and hardly any evidence for the importance of the fit between stereotype signals and job demands in personnel selection.

In sum, our results suggest that warmth and competence signals in application documents hardly affect individuals’ chances of receiving a positive response from the employer, while gender and ethnic group membership matter a lot. It is also possible that these characteristics worked as cues for the “Big Two,” implicating that warmth and competence played a more *indirect* role. As an example, and in line with previous findings on gender stereotypes (Fiske, 2017; Rudman & Glick, 1999), our validation study showed that women were in general perceived as warmer than men. Given the female premium we found in the main study, it might be that warmth actually did play a role in the hiring decisions, but not through self-accredited warmth in the application letter but through group membership and associated stereotypes. The hierarchy observed between the four origin groups also fits this explanation. The response rate is highest for warm and competent native followed by warm and competent European applicants, it is lower for competent but cold Asian applicants, and it is lowest for incompetent and cold MEA applicants.

In a similar vein, the null findings might be influenced by screening effects. While the information about applicants’ gender and ethnic origin is ascribed by the applicant’s name (which is most likely the first thing an employer notices), the information that the applicant is warm and competent only becomes evident at the end of the application through careful reading. However, if employers have very strong preferences, for example, in favor of native job applicants, he or she might never continue reading applications from a person with a foreign-sounding name and thus never find out that in addition to being an immigrant, the applicant is warm and competent. This reasoning is in line with findings from Bertrand and Mullainathan (2004), who point out the role of lexicographic search by employers who have scarce time resources and therefore use quick heuristics to sort out applications, for example, by the applicant’s name.

As gender and origin groups are central to people’s self-concept and such groups have virtually impermeable boundaries, there is no easy way to address the observed inequalities. Stressing warmth and competence in application documents is apparently not enough to equalize the hiring chances between members of different social groups. Moreover, our findings suggest that simply adding more information is not always a good idea: While appearing competent is never harmful (and our results suggest that it might benefit applicants), signaling warmth may even backfire. To reduce inequality in hiring, therefore, helping employers to focus on job-relevant characteristics might be a better strategy than adding more information. The formalization of application documents and procedures (at least at the first stage of the hiring process) might also be a promising strategy to reduce discrimination as it brings job-relevant characteristics—such as qualifications, skills, and experiences—to the fore, while detracting attention from irrelevant ones—such as gender and ethnic origin.

### Limitations and Future Directions

While providing unique empirical evidence on the (absence of strong) consequences of warmth and competence information in real world hiring decisions, our study has several limitations. Most importantly, we cannot rule out the possibility that the lack of evidence for the effect of signaling warmth (and for the interaction between stereotype signals and job demands or candidate characteristics, respectively) was due to a weak manipulation. Because we prioritized the ecological validity of our study over experimental power, we signaled warmth and competence only by means of a self-description, while we reinforced the self-description as competent with a list of additional job tasks and supervisory responsibilities in the resume—which is most likely a more objective indicator and therefore a more valid piece of information in the eyes of employers. Nevertheless, our signals of warmth and competence were rather unobtrusive. In addition, positive information about competence has been shown to be more diagnostic and influential than positive information about warmth, while negative warmth information is more diagnostic and thus influential than negative competence information (Cuddy et al., 2011; Tausch et al., 2007). As stereotype signals in this study were either positive or absent but never negative, our design might have masked the true penalty employers attach to indicators of a cold personality. It should be noted, however, that people engage in “impression management” in real hiring situations, as they are motivated to convince the employer that they are a good fit for the job.

This being said, the post hoc validation study described in the Online Supplement showed that the manipulation of warmth and competence did have significant effects on perceived communion and agency, suggesting that the manipulation of both signals worked as intended. However, reading an application as part of an online survey is different from screening applications in a noisy working environment. Employers are often under time pressure when making hiring decisions. They may use quick heuristics based on surface-level characteristics, such as age, gender, or ethnicity, while sifting through a pile of applications and fail to notice more subtle attributes like self-descriptions of warmth and competence. Even though we emphasized this information in both the cover letter and the CV, employers may have simply overlooked this information. We therefore cannot completely rule out that the experimental manipulation was in fact too weak (even if it was weak for good reasons) to have an effect. If so, the results of our study may underestimate the relevance that warmth and competence of applicants would have if employers took notice—which would become very likely in a job interview, for example.

Second, and related to the previous paragraph, this experiment only tests for the effect of signaling warmth and competence in the first step of the hiring process. In this step, decisions are formed based on application documents. Warmth, however, is a deep-level characteristic that is difficult to signal in application documents. Any statement about a warm personality is at risk of being understood as self-promotion, showing-off, or narcissism, which might contradict the stereotype of a truly warm and altruistic person. Importantly, while we think it is very likely that applicants can leave an even stronger warm impression during (face-to-face) interaction at the job interview based on cues such as eye contact, body language, voice tone, and so on (e.g., Rivera, 2015), our results show that ethnic minorities are more often denied this opportunity. We hope to encourage future studies with different designs to try to address the importance of warmth and competence signals in more dynamic job interview situations.

Third, our conceptualization of the stereotype-related signals only included the two dimensions warmth and competence described in classical literature, while more recent research found that these dimensions might have more specific facets (warmth and morality/competence and assertiveness; see Abele et al., 2016). In our validation study, we therefore considered the specific facets in addition to the two dimensions. The competence signal increased perceived agency and its two facets: assertiveness and competence. The warmth signal, by contrast, increased perceived communion as well as perceived warmth (the first facet) but not perceived morality (the second facet). Further research should address this point by using more nuanced warmth and competence signals.

Fourth and finally, while our results question the importance of warmth and competence signals in application documents, they highlight how group membership affects individual job candidates’ hiring chances. Despite equal qualifications, in our study, employers clearly favored female job candidates over male ones and showed ethnic discrimination, in particular against job candidates whose families originate in MEA countries. Our findings hopefully stimulate further research on the role of group stereotypes—in particular *ethnic* stereotypes—for discrimination in hiring.

## Research Ethics and Supporting Information

The authors verify that the data collection complied with ethical research principles. Each national research team successfully applied for ethics approval in their respective country. A technical report and the codebook of this study are publicly available (see Lancee, Birkelund, Coenders, Di Stasio, Fernández Reino, Heath, Koopmans, Larsen, Polavieja, Ramos, Soiné, et al., 2019; Lancee, Birkelund, Coenders, Di Stasio, Fernández Reino, Heath, Koopmans, Larsen, Polavieja, Ramos, Thijssen, et al., 2019). As the raw data contain sensitive information about real firms, they are not publicly available. Access to an anonymized data set can be provided on individual request.

## Supplemental Material

sj-pdf-1-psp-10.1177_0146167220982900 – Supplemental material for The “Big Two” in Hiring Discrimination: Evidence From a Cross-National Field Experiment Click here for additional data file. Supplemental material, sj-pdf-1-psp-10.1177_0146167220982900 for The “Big Two” in Hiring Discrimination: Evidence From a Cross-National Field Experiment by Susanne Veit, Hannah Arnu, Valentina Di Stasio, Ruta Yemane and Marcel Coenders in Personality and Social Psychology Bulletin

## Notes

1 First-generation immigrants are foreign-born immigrants. Second-generation immigrants are the descendants of first-generation immigrants, who are themselves born in the host country.

2 Not including Denmark, Ireland, and the Netherlands.

3 Maio et al. (1996) proposed an alternative hypothesis. They argued that ambivalent stereotypes would cause polarized responses. Employers might accordingly react more strongly to warmth or competence information about job applicants from immigrant groups with ambivalent stereotypes. That is, when stereotypes are positive and negative at the same time, additional information may have the power to change employer responses to the better.

4 A survey on the stock photos that we used confirmed that they were perceived as comparable in terms of attractiveness, competence, and warmth.

5 We grouped origin countries into broader world region clusters to reduce complexity. We acknowledge that stereotypes about specific countries might, to some extent, deviate from the stereotypes associated with broader world regions. For example, there is likely quite some variation *within* the European group in how competent and warm members of each single European country are perceived. As shown by Cuddy et al. (2009), Germans are stereotypically cold but competent, while Southern Europeans are rather warm but less competent. Nevertheless, European immigrants as a whole are typically perceived as more competent and warmer than immigrants from the Global South, and as comparable in warmth and competence with the in-group. A similar reasoning can be applied to other groups: For example, Japanese immigrants are regarded as more competent than Korean or Chinese, but the group of Asian immigrants as a whole has higher scores on the competence dimension than immigrants from Middle Eastern or African (MEA) countries (Lee & Fiske, 2006).

6 We use the terms warmth and competence in a broader sense because we had to adjust the operationalization of these theoretical concepts to the specific context under study.

7 It was essential for our study to create plausible applications that would not raise any suspicion by employers in different national contexts. As it would be very unusual for job applicants to present themselves negatively to employers, we decided to either add information that would make the candidates appear warm or competent, or not to include that information. Unlike Agerström et al. (2012), however, we did not add information that would make the candidates appear cold or incompetent. Second, we used self-descriptions instead of testimonials of others in the form of reference letters to ensure the ecological validity of our study, because including reference letters in job applications is not a common practice in some European countries, for example, the United Kingdom. Moreover, reference letters had no (Koopmans et al., 2019) or mixed effects (Kaas & Manger, 2012) in previous studies from Germany. Therefore, we opted for a manipulation with self-descriptions, which is perhaps subtle, but on the contrary more likely to reflect the application situation in the real world.

8 In the United Kingdom, where personal information in job applications is kept to a minimum, the applicants more generally referred to a willingness to relocate and stressed that, despite their foreign background (here the specific country of origin was mentioned), all their education and training had been completed domestically.

9 When replacing the two independent dummies for the warmth and competence signal with a four-level factor variable (0 = “no signal,” 1 = “warmth only,” 2 = “competence only,” and 3 = “warmth and competence”), the effect of signaling competence became stronger (*b* = .02, *p* < .05) while signaling warmth or warmth and competence had no effect (*b* = .01, *ns*, respectively).

10 For this analysis, we used the test-command ins Stata (“test warmth=competence”) after the regressions shown in Online Supplement Table S2:1. Adding analytical weights did not change this result. Moreover, the effect of signaling “competence only” was not significantly different from the effect of signaling “warmth only”: *b* = .01, *ns.* (see Note 8).
