# Supplementary material for: The “Big Two” in Hiring Discrimination: Evidence From a Cross-National Field Experiment
Source: Pers Soc Psychol Bull. 2021 Mar 6;48(2):167–82. doi: 10.1177/0146167220982900 (PMC8801666; doi:10.1177/0146167220982900)
Supplement: sj-pdf-1-psp-10.1177_0146167220982900 – Supplemental material for The “Big Two” in Hiring Discrimination: Evidence From a Cross-National Field Experiment [file sj-pdf-1-psp-10.1177_0146167220982900.pdf]

Online supplement for

The „Big Two“ in hiring discrimination: Evidence from a cross-national field experiment

Susanne Veit[1,2] , Hannah Arnu[1], Valentina Di Stasio[3],

Ruta Yemane[1,2], and Marcel Coenders[4]

[1]DeZIM-Institut, Berlin, Germany
[2]WZB Berlin Social Science Center, Berlin, Germany
[3]Utrecht University, Utrecht, The Netherlands
[4]Sociaal en Cultureel Planbureau (SCP), Den Haag, The Netherlands

I. Supplementary Tables & Figures

Table S1: Descriptive of main variables

Variables				Obs	%	Min	Max
**DEPENDENT VARIABLE**							
			Positive employer response	13,162	31.9	0	1
**TREATMENT VARIABLES**							
Stereotype signal			Warmth	13,162	50.8	0	1
			Competence	13,162	50.3	0	1
Origin group			Native	13,162	30.1	0	1
			European	13,162	22.8	0	1
			Asian	13,162	16.9	0	1
			MEA	13,162	30.2	0	1
Gender			Male	13,162	51.6	0	1
Job demands:	High-skilled	Customer contact					
Occupation	no	no	Cook	13,162	21.6	0	1
	no	no	Payroll Clerk	13,162	20.3	0	1
	no	yes	Receptionist	13,162	12.2	0	1
	yes	yes	Sales Representative	13,162	16.4	0	1
	yes	no	Software Developer	13,162	14.1	0	1
	no	yes	Store Assistant	13,162	15.4	0	1
**CONTROL VARIABLES**							
Religion			No religion	13,162	50.5	0	1
			Christian	13,162	30.2	0	1
			Muslim	13,162	16.8	0	1
			Buddhist	13,162	2.3	0	1
			Hindu	13,162	0.3	0	1
Grade information			yes	13,162	50.8	0	1
Country			Germany	13,162	18.8	0	1
			Netherlands	13,162	25.0	0	1
			Norway	13,162	12.3	0	1
			Spain	13,162	22.0	0	1
			United Kingdom	13,162	21.8	0	1

Table S2: Linear regression of employer responses on experimental treatments, job characteristics, and countries

**DV: Positive response**		(1)	(2)	(3)	(4)	(5)	(6)	(7)	(8)
Warmth (ref: no)		.000	.010	.002	.000	-.015	-.000	-.008	-.000
		(.008)	(.011)	(.011)	(.008)	(.014)	(.008)	(.010)	(.008)
Competence (ref: no)		**.014***	**.024****	**.014***	.009	**.014***	.023	**.014***	.010
		(.008)	(.011)	(.008)	(.011)	(.008)	(.014)	(.008)	(.009)
Origin group (ref: native)	European	**-.038*****	**-.038*****	**-.038*****	**-.038*****	**-.047*****	**-.027***	**-.038*****	**-0.038*****
		(.011)	(.011)	(.011)	(.011)	(.016)	(.015)	(.011)	(0.011)
	Asian	**-.066*****	**-.066*****	**-.066*****	**-.066*****	**-.075*****	**-.059*****	**-.066*****	**-0.066*****
		(.013)	(.013)	(.013)	(.013)	(.017)	(.017)	(.013)	(0.013)
	MEA	**-.094*****	**-.094*****	**-.094*****	**-.094*****	**-.106*****	**-.091*****	**-.094*****	**-0.094*****
		(.011)	(.011)	(.011)	(.011)	(.015)	(.015)	(.011)	(0.011)
Male (ref: female)		**-.049*****	**-.049*****	**-.047*****	**-.054*****	**-.049*****	**-.049*****	**-.049*****	**-.049*****
		(.008)	(.008)	(.011)	(.011)	(.008)	(.008)	(.008)	(.008)
High-skilled (ref: low-med)		**.132*****	**.133*****	**.132*****	**.132*****	**.132*****	**.132*****	**.132*****	**.125*****
		(.009)	(.009)	(.009)	(.009)	(.009)	(.009)	(.009)	(.012)
Customer contact (ref: little)		**-.129*****	**-.129*****	**-.129*****	**-.129*****	**-.129*****	**-.129*****	**-.138*****	**-.129*****
		(.008)	(.008)	(.008)	(.008)	(.008)	(.008)	(.011)	(.008)
**INTERACTIONS**			Warmth x Competence	Warmth x Male	Competence x Male	Warmth x Origin group	Competence x Origin group	Warmth x Customer	Competence x High-skilled
			-.019	-.004	.010			.018	.014
			(.015)	(.015)	(.015)			(.015)	(.017)
	*European					.018	-.022		
						(.022)	(.022)		
	*Asian					.018	-.015		
						(.023)	(.023)		
	*MEA					.025	-.006		
						(.020)	(.020)		
Intercept		.265***	.260***	.264***	.268***	.273***	.260***	.269***	.267***
		(.014)	(.015)	(.015)	(.015)	(.015)	(.015)	(.015)	(.014)
Obs.		13,162	13,162	13,162	13,162	13,162	13,162	13,162	13,162

Standard errors are in parenthesis
*** p<.01, ** p<.05, * p<.10 (two-tailed)
All estimates result from linear regression models with robust standard errors and controls for religion, grades, and study country.

Table S3: Probit regression

DV: Positive response		(1)	(2)	(3)	(4)	(5)	(6)	(7)	(8)
Warmth (ref: no)		.002	.034	.007	.002	-.038	.002	-.022	.002
		(.024)	(.034)	(.034)	(.024)	(.042)	(.024)	(.031)	(.024)
Competence (ref: no)		**.044***	**.077****	**.044***	.027	**.044***	**.072***	**.044***	.034
		(.024)	(.034)	(.024)	(.034)	(.024)	(.042)	(.024)	(.029)
Origin group (ref: native)	European	**-.114*****	**-.114*****	**-.114*****	**-.114*****	**-.137*****	**-.081***	**-.114*****	**-.114*****
		(.033)	(.033)	(.033)	(.033)	(.047)	(.046)	(.033)	(0.033)
	Asian	**-.206*****	**-.206*****	**-.206*****	**-.206*****	**-.233*****	**-.183*****	**-.206*****	**-.206*****
		(.041)	(.041)	(.041)	(.041)	(.056)	(.055)	(.041)	(0.041)
	MEA	**-.287*****	**-.287*****	**-.287*****	**-.287*****	**-.323*****	**-.279*****	**-.287*****	**-.287*****
		(.035)	(.035)	(.035)	(.035)	(.047)	(.047)	(.035)	(0.035)
Male (ref: female)		**-.154*****	**-.154*****	**-.149*****	**-.171*****	**-.155*****	**-.154*****	**-.154*****	**-.154*****
		(.024)	(.024)	(.034)	(.034)	(.024)	(.024)	(.024)	(.024)
High-skilled (ref: low-med)		**.392*****	**.393*****	**.392*****	**.393*****	**.392*****	**.392*****	**.392*****	**.376*****
		(.026)	(.026)	(.026)	(.026)	(.026)	(.026)	(.026)	(.037)
Customer contact (ref: little)		**-.401*****	**-.401*****	**-.401*****	**-.401*****	**-.400*****	**-.401*****	**-.430*****	**-.401*****
		(.025)	(.025)	(.025)	(.025)	(.025)	(.025)	(.035)	(.025)
**INTERACTIONS**			Warmth x Competence	Warmth x Male	Competence x Male	Warmth x Origin group	Competence x Origin group	Warmth x Customer	Competence x High-skilled
			-.064	-.009	.035			.058	.033
			(.048)	(.048)	(.048)			(.048)	(.051)
	*European					.045	-.065		
						(.065)	(.065)		
	*Asian					.053	-.047		
						(.073)	(.073)		
	*MEA					.072	-.016		
						(.061)	(.061)		
Intercept		-.684***	-.701***	-.687***	-.676***	-.663***	-.698***	-.672***	-.679***
		(.046)	(.047)	(.047)	(.047)	(.049)	(.049)	(.047)	(.046)
Obs.		13,162	13,162	13,162	13,162	13,162	13,162	13,162	13,162

Standard errors are in parenthesis
*** p<.01, ** p<.05, * p<.10 (two-tailed)
All estimates result from linear regression models with robust standard errors and controls for religion, grades, and study country.

Table S4: Logistic regression

DV: Positive response		(1)	(2)	(3)	(4)	(5)	(6)	(7)	(8)
Warmth (ref: no)		.003	.055	.014	.004	-.065	.003	-.037	.003
		(.040)	(.057)	(.056)	(.040)	(.070)	(.040)	(.052)	(.040)
Competence (ref: no)		**.074***	**.125****	**.073***	.044	**.073***	.115	**.073***	.054
		(.040)	(.057)	(.040)	(.056)	(.040)	(.070)	(.040)	(.049)
Origin group (ref: native)	European	**-.188*****	**-.188*****	**-.187*****	**-.188*****	**-.230*****	**-.134***	**-.188*****	**-.187*****
		(.054)	(.054)	(.054)	(.054)	(.077)	(.077)	(.054)	(.054)
	Asian	**-.349*****	**-.349*****	**-.350*****	**-.349*****	**-.392*****	**-.316*****	**-.350*****	**-.349*****
		(.070)	(.070)	(.070)	(.070)	(.094)	(.094)	(.070)	(.070)
	MEA	**-.481*****	**-.481*****	**-.481*****	**-.481*****	**-.544*****	**-.472*****	**-.482*****	**-.481*****
		(.059)	(.059)	(.059)	(.059)	(.079)	(.079)	(.059)	(.059)
Male (ref: female)		**-.259*****	**-.260*****	**-.247*****	**-.289*****	**-.260*****	**-.259*****	**-.259*****	**-.259*****
		(.040)	(.040)	(.057)	(.057)	(.040)	(.040)	(.040)	(.040)
High-skilled (ref: low-med)		**.660*****	**.660*****	**.660*****	**.660*****	**.659*****	**.659*****	**.660*****	**.630*****
		(.044)	(.044)	(.044)	(.044)	(.044)	(.044)	(.044)	(.061)
Customer contact (ref: little)		**-.680*****	**-.680*****	**-.680*****	**-.680*****	**-.679*****	**-.681*****	**-.731*****	**-.680*****
		(.042)	(.042)	(.042)	(.042)	(.042)	(.042)	(.059)	(.042)
**INTERACTIONS:**			Warmth x Competence	Warmth x Male	Competence x Male	Warmth x Origin group	Competence x Origin group	Warmth x Customer	Competence x High-skilled
			-.101	-.023	.059			.101	.058
			(.080)	(.080)	(.080)			(.082)	(.085)
	*European					.084	-.108		
						(.108)	(.108)		
	*Asian					.084	-.068		
						(.124)	(.124)		
	*MEA					.124	-.020		
						(.103)	(.103)		
Intercept		-1.152***	-1.179***	-1.158***	-1.137***	-1.115***	-1.172***	-1.130***	-1.142***
		(.078)	(.081)	(.081)	(.080)	(.084)	(.083)	(.080)	(.079)
Obs.		13,162	13,162	13,162	13,162	13,162	13,162	13,162	13,162

Standard errors are in parenthesis
*** p<.01, ** p<.05, * p<.10 (two-tailed)
The logistic regression models all control for religion, grades, and study country.

Table S5: Linear regression with analytical weights adjusting for cell size differences between origin country by study country and occupation

**DV: Positive response**		(1)	(2)	(3)	(4)	(5)	(6)	(7)	(8)
Warmth (ref: no)		-.008	.016	.001	-.008	**-.028***	-.008	-.026	-.008
		(.012)	(.017)	(.018)	(.012)	(.017)	(.012)	(.017)	(.012)
Competence (ref: no)		.004	.029	.004	.010	.004	.027	.003	-.001
		(.012)	(.018)	(.012)	(.018)	(.012)	(.017)	(.012)	(.014)
	European	**-.039*** **	**-.039*** **	**-.039*** **	**-.039*** **	**-.048** **	-.028	**-.039*** **	**-.039*** **
		(.013)	(.013)	(.013)	(.013)	(.019)	(.018)	(.013)	(.013)
Origin group (ref: native)	Asian	**-.068*** **	**-.068*** **	**-.068*** **	**-.068*** **	**-.079*** **	**-.070*** **	**-.069*** **	**-.069*** **
		(.018)	(.018)	(.018)	(.018)	(.025)	(.023)	(.018)	(.018)
	MEA	**-.096*** **	**-.095*** **	**-.095*** **	**-.096*** **	**-.107*** **	**-.072*** **	**-.096*** **	**-.096*** **
		(.016)	(.016)	(.016)	(.016)	(.022)	(.022)	(.016)	(.016)
Male (ref: female)		**-.049*** **	**-.050*** **	**-.040** **	**-.043** **	**-.049*** **	**-.050*** **	**-.049*** **	**-.049*** **
		(.012)	(.012)	(.018)	(.017)	(.012)	(.012)	(.012)	(.012)
High-skilled (ref: low-med)		**.146*** **	**.146*** **	**.146*** **	**.146*** **	**.146*** **	**.146*** **	**.146*** **	**.139*** **
		(.014)	(.014)	(.014)	(.014)	(.014)	(.014)	(.014)	(.020)
Customer contact (ref: little)		**-.118*** **	**-.118*** **	**-.118*** **	**-.118*** **	**-.118*** **	**-.118*** **	**-.137*** **	**-.118*** **
		(.012)	(.012)	(.012)	(.012)	(.012)	(.012)	(.018)	(.012)
**INTERACTIONS**			Warmth x Competence	Warmth x Male	Competence x Male	Warmth x Origin group	Competence x Origin group	Warmth x Customer	Competence x High-skilled
			-.049**	-.018	-.013			.037	.015
			(.025)	(.025)	(.025)			(.025)	(.028)
	*European					.019	-.022		
						(.026)	(.026)		
	*Asian					.022	.003		
						(.031)	(.031)		
	*MEA					.023	**-.047***		
						(.028)	(.028)		
Intercept		.274***	.260***	.269***	.271***	.284***	.263***	.284***	.277***
		(.022)	(.023)	(.023)	(.023)	(.023)	(.022)	(.023)	(.022)
Obs.		13,162	13,162	13,162	13,162	13,162	13,162	13,162	13,162

Standard errors are in parenthesis

*** p<.01, ** p<.05, * p<.10 (two-tailed)

All estimates result from linear regression models with robust standard errors, analytical weights (that adjust for differences in the number of observations by origin group, country, and occupations), and controls for religion, grades, and study country.

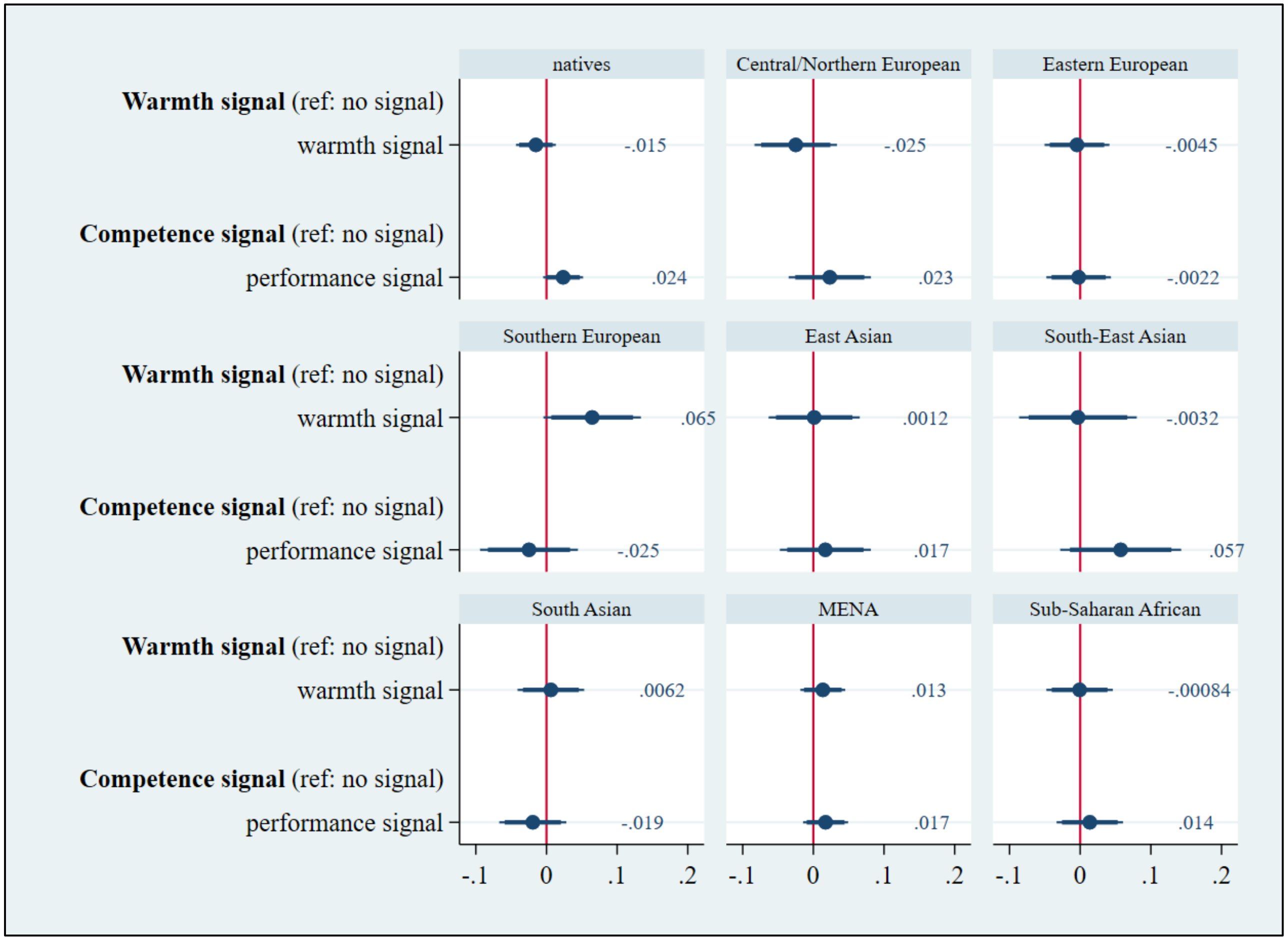

Figure S1: Results of separate regressions with more fine-grained regional origin groups

These coefficients plots show the effects (with 90% and 95% confidence intervals) of warmth and competence signals on response rates in separate linear regressions for more fine-grained regional origin groups. The following origin countries belong to each group: Central and Northern Europe: France, Germany, Netherlands, Norway & United Kingdom (not for natives, respectively); Southern Europe: Greece, Italy & Spain (the latter not in Spain); Eastern Europe: Albania, Bulgaria, Poland, Romania & Russia; East Asia: China, Japan & South-Korea; South-East Asia: Indonesia & Vietnam; South Asia: India & Pakistan; MENA: Egypt, Iran, Iraq. Lebanon, Morocco & Turkey; and Sub-Saharan Africa: Ethiopia, Nigeria, & Uganda. All estimates result from linear regressions models with robust standard errors and controls for job candidates' religion, grades, and the country of study.

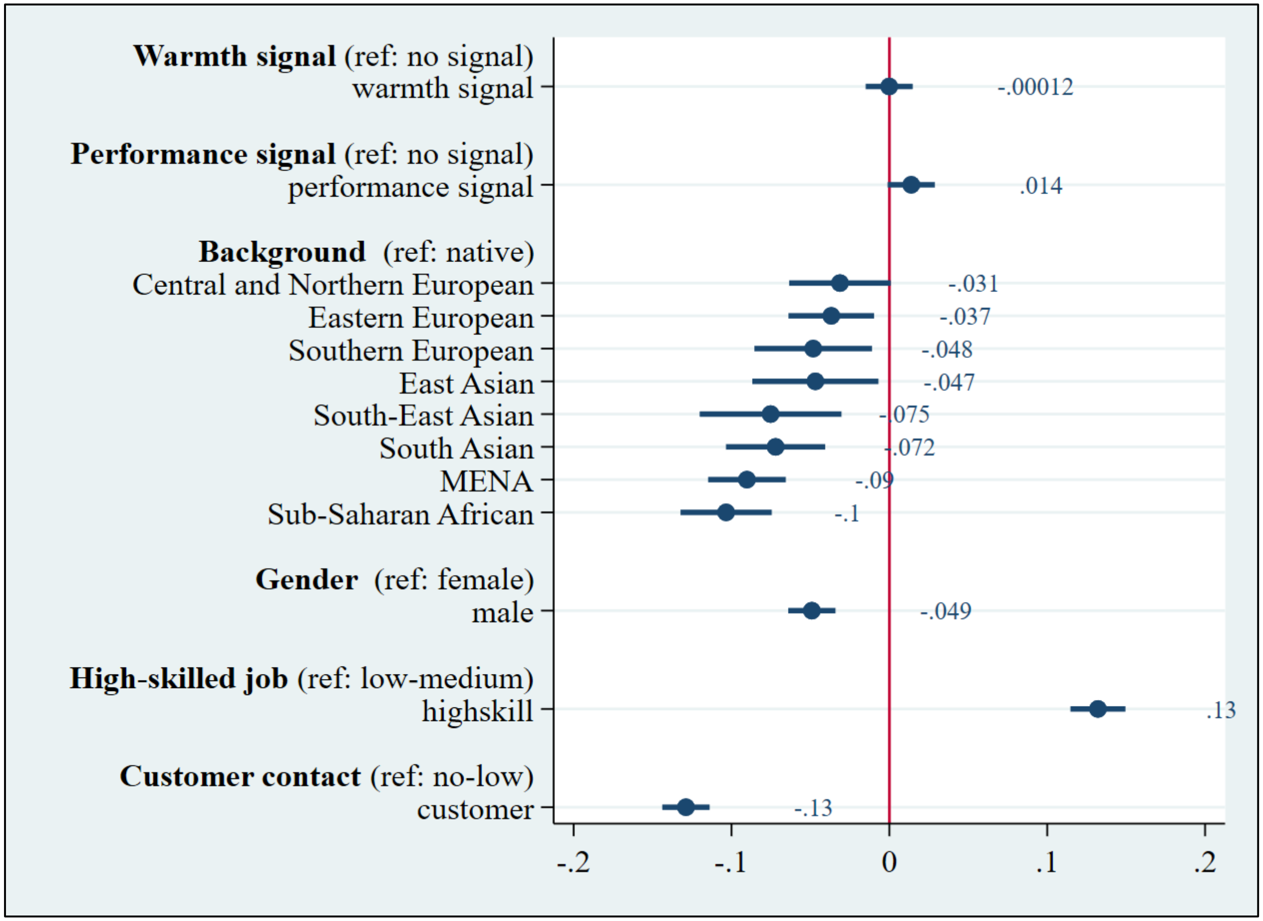

Figure S2: Main effects for more fine-grained regional origin groups

This coefficients plot shows the main effects from the linear regression (with robust standard errors and controls for job candidates' religion, grades, and the country of study) of employer response on the independent variables (with 90% and 95% confidence intervals). The analysis considers more fine-grained regional origin groups. The following origin countries belong to each group: Central and Northern Europe: France, Germany, Netherlands, Norway & United Kingdom (not for natives, respectively); Southern Europe: Greece, Italy & Spain (the latter not in Spain); Eastern Europe: Albania, Bulgaria, Poland, Romania & Russia; East Asia: China, Japan & South Korea; South-East Asia: Indonesia & Vietnam; South Asia: India & Pakistan; MENA: Egypt, Iran, Iraq. Lebanon, Morocco & Turkey; and Sub-Saharan Africa: Ethiopia, Nigeria & Uganda.

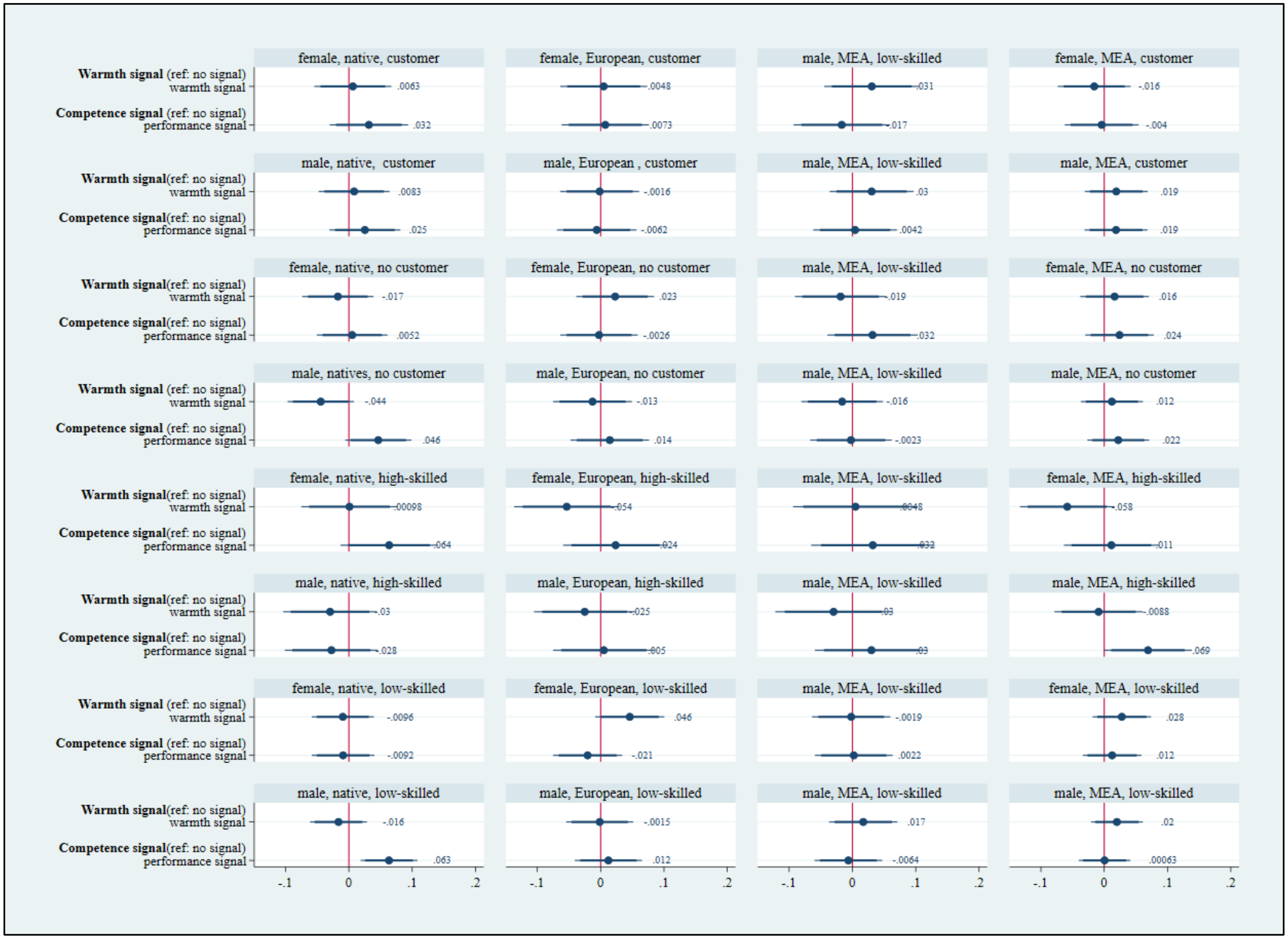

Figure S3: The effect of warmth and competence signals for males and females of different origin in jobs with different demands

These coefficients plots show the effects (with 90% and 95% confidence intervals) of warmth and competence signals on response rates in separate linear regressions for male and female job candidates of different origin in occupations with low or high levels of customer contact or qualification requirements, respectively. All estimates result from linear regressions models with robust standard errors and controls for job candidates' religion, grades, and study country.

II. Validation study

As proposed by the anonymous reviewers of our manuscript and the editor, we conducted a post-hoc validation study to test whether the warmth and competence manipulation in the application documents increased, as intended, the perceived warmth and competence of the job applicant.

Design and method

The post-hoc validation study was conducted with a German convenience sample. The online survey was advertised on Facebook in groups that are predominantly used by German (psychology) students. Data were gathered between March and April 2020 [1]. 318 users participated in the validation study (M_{Age} = 26.3, SD_{Age} = 6.23; 71 % female).

In this study, each respondent was randomly assigned to one job application. The application documents consisted of a cover letter and a CV with photo. In contrast to the main study, we did not show copies of school leaving or job training certificates to reduce the number of documents and the amount of information; in particular, we expected some people to fill in the survey on a mobile phone and copies of certificate would not have been easily readable. However, we informed respondents that this information had also been provided by the job candidate. After reading the application documents, respondents were asked to evaluate the job candidate with respect to warmth and competence.

Experimental treatments

We used four profiles from the main study: male and female applicants (with typical German names and photos, i.e. white skin and brown hair) that applied for two medium-skilled jobs, one with little (payroll clerk) and one with rather frequent customer contact (hotel receptionist). In addition, we randomly assigned the warmth and competence treatments from the main study.

[1] In March and April 2020 German society was increasingly affected by restrictions on public life as a means to combat the Corona pandemic. To test for potential biases in consequence of this extraordinary situation, we tested for time effects; but there were no significant changes over time.

Each respondent was randomly assigned to one out of the resulting 16 (gender by job by warmth by competence: 2-2-2-2 design) profiles.

Dependent variables

We asked respondents to evaluate warmth and competence using the items from the four facets of agency and communion that had been proposed by Abele et al. (2016)[2], asking the participants to rate how much the respective traits applied to the applicant. A bipolar format with a 5-point Likert-scale (e.g., from "little efficient" to "very efficient") was used for each of the 20 items. The scale considers more recent research on the Big Two suggesting two facets of communion (warmth and morality) and two facets of agency (competence and assertiveness). Table S6 further below lists all items in English and in German.

Results

To test whether the four dimensions proposed by Abele et al. (2016) emerge, as the first step we conducted an exploratory factor analysis (EFA) with maximum likelihood estimation and maximum *four* factors to be retained on the 10 communion and the 10 agency items with. This yielded two factors with eigenvalues greater than one, which explained 72 and 18 percent of the variance, respectively (Schwarz's *BIC* = 627.987, *AIC* = 352.442). Thereafter, we repeated this analysis with maximum *three* factors to be retained. Again, two factors with eigenvalues greater than one emerged. They explained 76 and 19 percent of the variance, respectively (Schwarz's *BIC* = 595.549, *AIC* = 383.305). Thereafter, we run an EFA with maximum *two* factors to be retained, now explaining 80 and 20 percent of the variance (Schwarz's BIC = 561.665, AIC = 416.446). The

[2] Abele, A. E., Hauke, N., Peters, K., Louvet, E., Szymkow, A., & Duan, Y. (2016). Facets of the fundamental content dimensions: Agency with competence and assertiveness—Communion with warmth and morality. *Frontiers in psychology, 7*, 1810. https://doi.org/10.3389/fpsyg.2016.01810

scree-test also yielded two factors with eigenvalues larger than one. Using R, we did a parallel analysis, which also suggested a two-factor solution.

Next, we investigated the rotated factor solutions. An orthogonal varimax rotation of the two-factor solution yielded a very conclusive pattern of factor loadings (see Table S6), while the rotated factor loadings in the four-factor solution did not provide a meaningful pattern.[3] All items from the two facets of agency, that is assertiveness (AA1-5) and competence (AC1-5), and two items from the morality facet of communion (CM4 & CM5) loaded on the first factor with values larger than .40 (except for AA5: loading <.40). The second factor was comprised of all ten communion items with factor loading larger than .40, including the warmth (CW1-5) and the morality (CM1-5) facets. We excluded the two morality items that loaded highly on both factors (CM4 & CM5) and the assertiveness item (AA5) with loadings below .40 from the two factors. The rotated factors explained 51 and 49 percent of the variance.

Table S6: Rotated factor loadings

Item	Factor 1	Factor 2	Uniqueness
CW1		0.7709	0.3592
CW2		0.6217	0.5619
CW3		0.7117	0.4654
CW4		0.7659	0.3889
CW5		0.4938	0.6475
CM1		0.6399	0.5536
CM2		0.5950	0.5959
CM3		0.6538	0.5259
CM4	*0.4376*	*0.4880*	0.5703
CM5	*0.5389*	*0.4480*	0.5089
AA1	0.6392		0.5843
AA2	0.6660		0.5353
AA3	0.4768		0.7439
AA4	0.6092		0.6269
AA5			0.8506
AC1	0.6961		0.4621
AC2	0.6702		0.4639
AC3	0.7207		0.3614
AC4	0.5956		0.5113
AC5	0.6683		0.4694

Note: Exploratory factor analysis with maximum two factors to be retained (maximum likelihood method, orthogonal Varimax rotation). Blanks represent loadings smaller than .30 and Items with cross-loadings are highlighted in italics.

[3] The third and fourth factors contained only one item with loadings larger than .40, respectively: AA5 and AC5.

As a second test of the factorial structure of the data, we ran confirmatory factor analyses (CFA) with all items. The fit indices of both the two– and the four factor solution were acceptable to good (except for chi2, which, however, tends to fail for large samples), but they were overall slightly better for the four-factor solution (*RMSEA*=.072, *CFI*=.904, *SRM*R=.062, *chi2*(164)=427.63, *p*<.001) compared to the two-factor solution (*RMSEA*=.079, *CFI*=.883, *SRMR*=.072, *chi2*(169)=491.28, *p*<.001).[4]

In sum, our explanatory factor analyses recommended a two-factor solution, while confirmatory factor analyses were slightly more supportive of the four factor solutions, which ties in with the facets model that was developed by Abele et al. (2016). Both models fit the data quite well. Since the decision of whether the facets model or classic Big-Two models are better suited to describe fundamental stereotype content dimensions is beyond the scope of this validation study, we computed in total six scales to test the validity of our experimental material: four scales that reflect the facets model (i.e. *Warmth, Morality, Assertiveness*, and *Competence*) and two scales that combine the facets (i.e. *Communion* and *Agency*)[5]. The internal consistency of all six scales was high (see Table S7). On average, job applicants were rated positively on all four facets and on the two fundamental dimensions; with values slightly above 3.5 on response scales from 1 to 5 (see Table S7).

---

[4] When limiting the analyses to the 17 items from Table S6 with high loading and no cross-loading, the fit indices further improved - but the four-factor solution had again a slightly better fit (four-factor solution: *RMSEA* = .064, *CFI* = .939, *SRMR* = .046, *Chi2*(118)=255.410, *p*<.001; two-factor solution: *RMSEA* = .067, *CFI* = .930, *SRMR* = .053; *Chi2*(118)=283.023, *p*<-001)

[5] In the mains study, we termed these two scales perceived "warmth" and "competence" instead of "communion" and "agency" to be more consistent with the designation of the experimental treatments.

Table S7: Stereotype content items

**Communion**				**Agency**			
**Warmth**		**Morality**		**Assertiveness**		**Competence**	
5 items		*5items*		*5 items*		*5 items*	
α = .84		*α* = .79		*α* = .70		*α* = .86	
M = 3.61, *SD* = .65		*M* = 3.66, *SD* = .60		*M* = 3.46, *SD* = .63		*M* = 3.71, *SD* = .69	
CW1 little caring – very caring	wenig fürsorglich – sehr fürsorglich	CM1 unjust - just	ungerecht - gerecht	AA1 not at all self-confident – very self-confident	nicht selbstsicher – sehr selbstsicher	AC1 little efficient - very efficient	wenig effizient - sehr effizient
CW2 very cold in relations with others – very warm in relations with others	sehr kühl in Beziehungen zu anderen – sehr herzlich in Beziehungen zu anderen	CM2 little fair - very fair	wenig fair - sehr fair	AA2 go to pieces under pressure – stand up well under pressure	kann Druck nicht standhalten – kann Druck gut standhalten	AC2 little capable - very capable	wenig leistungsfähig - sehr leistungsfähig
CW3 little empathetic – very empathetic	wenig einfühlsam – sehr einfühlsam	CM 3very inconsiderate - very considerate	sehr rücksichtslos - sehr rücksichtsvoll	AA3 give up very easily – never give up easily	gibt leicht auf – gibt nie leicht auf	AC3 little competent - very competent	wenig kompetent - sehr kompetent
CW4 little affectionate - very affectionate	wenig liebevoll - sehr liebevoll	~~CM4 not trustworthy – very trustworthy~~	~~nicht vertrauenswürdig – sehr vertrauenswürdig~~	AA4 have no leadership abilities at all - have leadership qualities	ist kein Führungstyp - hat Führungseigenschaften	AC4 little intelligent - very intelligent	wenig intelligent - sehr intelligent
CW5 not at all friendly – very friendly	sehr unfreundlich – sehr freundlich	~~CM5 not very reliable - very reliable~~	~~wenig zuverlässig - sehr zuverlässig~~	~~AA5 feel very inferior – feel very superior~~	~~fühlt sich unterlegen – fühlt sich überlegen~~	AC5 little clever – very clever	nicht clever – sehr clever
**Communion**				**Agency**			
8 items				9 items			
α = .88				*α* = .88			
M = 3.55, *SD* = .61				*M* = 3.65, *SD* = .65			

Exploratory factor analyses, by contrast, yielded consistently two factors with eigenvalues larger than one. In the rotated two-factor solution (orthogonal, varimax), two out of the five morality items had high cross-loadings (i.e. they loaded strongly on both factors) and the loading of one assertiveness item was weaker than .40 (see Table S6). We therefore excluded these three items (see the ~~crossed~~ items) und summarized the remaining 17 items to two scales: Communion and Agency. Because the results of confirmatory factor analyses were slightly in favor of a four-factor solution, we in addition provide the descriptive statistics for the four facets scales: Warmth, Morality, Assertiveness, and Competence.

The results confirmed that applicants who described themselves as warm were indeed rated higher in communion (*b*=.13, *p*<.10) and warmer (*b*=.13, *p*<.10) than applicants who did not include this information in the application (see the first two columns in row A of Table S8). For the morality facet there was no effect (*b*=.05, *ns*); which makes intuitive sense, since the warmth treatment in the application documents did not reflect morality but a warm and social personality. Neither adding the competence treatment (row B) nor adding the competence treatment, applicants' gender, and the job (row C) changed the results.

S8: Linear regression results

			Facets			*Facets*		
		**Communion**	***Warmth***	***Morality***	**Agency**	***Assertiveness***	***Competence***	
**A**	**Warmth treatment**: yes (vs. no)	**.13 (.07)+**	**.13 (.07)+**	.05 (.07)	**.25(.07)****	**.29 (.07)*****	**.19 (.08)***	**Competence treatment:** yes (vs. no)
**B**	**Warmth treatment**: yes (vs. no)	**.13 (.07)+**	**.13 (.07)+**	.05 (.07)	-.01 (.07)	.02 (.07)	-.02 (.08)	Warmth treatment: yes (vs. no)
	Competence treatment: yes (vs. no)	.08 (.07)	.10 (.07)	.11 (.07)	**.25 (.07)****	**.29 (.07)*****	**.19 (.07)***	**Competence treatment:** yes (vs. no))
**C**	**Warmth treatment**: yes (vs. no)	**.13 (.07)+**	**.13 (.07)+**	.05 (.07)	-.01 (.07)	.01 (.07)	-.02 (.08)	Warmth treatment: yes (vs. no)
	Competence treatment: yes (vs. no)	.07 (.07)	.09 (.07)	.10 (.07)	**.24 (.07)****	**.29 (.07)*****	**.19 (.08)***	**Competence treatment**: yes (vs. no))
	Applicant's gender: female (vs. male)	**.18 (.07)****	**.21 (.07)****	.09 (.07)	.06 (.07)	-.04 (.07)	.13 (.08)	Applicant's gender: female (vs. male)
	Job: Payroll clerk (vs. Hotel receptionist)	-.09 (.07)	-.10 (.07)	-.09 (.07)	-.09(.07)	-.11(.07)	-.05(.08)	Job: Payroll clerk (vs. Hotel receptionist)
	N	318	318	318	318	318	318	
	R^2	.01-.03	.01-.03	.00-.01	.03	.05	.01-.02	

Standard errors are in parenthesis
*** p<.001, ** p<.01,* p<.05, + p<.10 (two-tailed)

In a similar vein, applicants who described themselves as competent were also perceived as more agentic (b=.25, p<.01), assertive (b=.29, p<.001), and competent (b=.19, p<.05) than applicants who did not include this information in the application (see row A on the right-hand side of Table S8). Again, these effects hardly changed when simultaneously controlling for the warmth treatment (row B) or the warmth treatment, applicants' gender, and the job to the models (row C). Importantly, the competence treatment had no effect on perceived communion, warmth, or morality, and the warmth treatment had no effect on perceived agency, assertiveness, or competence.

In line with previous studies in gender stereotypes, we confirmed that female job applicants were generally (and in line with gender stereotypes) perceived as being warmer and higher in communion than male job applicants—independent of the absence or presence of the warmth treatment. Warmth and competence ratings did not vary systematically between job types.

Summary and Discussion

In sum, the validation study confirmed that both the warmth and the competence treatments had the intended effects. However, the effect of the warmth treatment was only marginally significant (i.e., significant with a directed hypothesis) and both effects —i.e. the effect of the warmth treatment and the effect of the competence treatments—were rather small in terms of effect size.

The low power of our manipulation is of course a potential problem for the interpretation of null-findings in the main study, because it might imply either that the hypothesis was false or the manipulation too weak. However, there was hardly any alternative. Our experimental material was created for a large-scale field experiment on the discrimination of ethnic minorities in the labor market in five European countries, which means that the plausibility of the experimental material (the applicant had to be a real competitor for the advertised position and it was important to avoid the risk of detection) and the comparability across national contexts (e.g., while in all countries under study self-reports are a typical component of applications documents,

reference letters are unusual for the UK but usual for Germany) were of highest priority. Otherwise the findings of such a study would be misleading. The use of excessively strong, artificial, or unusual experimental treatments could bias the observable between-group and between-country results in either direction. The priority of our study was to create carefully constructed, plausible and comparable application documents, which comes at the cost of having rather unobtrusive warmth and competence signals and thus a rather weak experimental manipulation. The significant impact of the warmth and competence signals on candidates' assessment, however, suggests that we succeeded in finding the right balance between signal strength and plausibility.
